# False Impressions

**Published:** 1888-11-10

**Authors:** Caroline Fothergill

**Affiliations:** Author of "An Enthusiast," "A Voice in the Wilderness," etc.


### FALSE IMPRESSIONS.

By CAROLINE FOTHERGILL, Author of "An Enthusiast," "A Voice in the Wilderness," etc.

*(Continued from p. 78.)*

### CHAPTER VI.—EDITH.

ALL this time George was almost unconscious that his house contained such an inmate as Edith Lester, and Edith herself was getting used to the idea that Mayfield was to be her home. The prospect had no great charm for her. Her home in London had, compared with the house in Queen Street, been poor and rough to an extraordinary degree. Edith, as the only girl of the family, with half-a-dozen tumultuous brothers to look after, had had no easy time of it; yet her brothers were very fond of her. They had petted and spoiled her as much as they knew how, and although they had offered no serious opposition to her going to stay with her aunt Frances, they entirely disapproved of her going to live there altogether. The younger ones simply called it "a beastly shame"; the elder resented it as the withdrawal of the last gleam of civilisation from their home. But their father was firm. Edith's future had been a source of great anxiety to him. As long as he lived she could stay under his roof, her home was assured; but when he died—and he was neither a young nor a strong man, his heart failed him at the prospect. Edith, little, delicate, timid Edith, could not, should not go out among strangers to earn her bread as a governess, and which of her brothers would be able to offer her a home? They would marry, and then Edith would once more be thrown adrift. Of course Edith might marry too, but in her present position the husband who would fall to her share could only be a poor curate, or some fellow-clerk of her brothers, and he would rather she never knew the meaning of love than made such a marriage, and went through the old drudgery which had hastened her mother into her grave. Therefore, when Mrs. Lester's letter came, asking that Edith might stay with her permanently, he had smothered his yearning for the company of his only daughter, and had consented to the arrangement with her rich aunt. Her home was safe, and if she married she stood a better chance of marrying comfortably.

Edith herself acquiesced in the arrangement with her natural docility. She suddenly found out how very dear to her were her father and "the boys;" but she was accustomed to doing as she was told, and after some tears, and longing for the old rough home, with the incessant tramp of boys' feet and clamour of boys' voices, its worn-out carpets and furniture, and plain, frugal fare, she settled down to the peace and plenty of Queen Street, the noiselessly-moving servants, and the quiet perfection of all the appointments. These things had oppressed her at first, then she had grown used to them, now that she knew her life was to be passed amidst them they oppressed her again, and she moved and spoke with even more than her usual quietness and shyness.

She certainly was not an obtrusive guest, gentle and mouse-like in her movements, chary of her speech to this brother of her aunt. He often forgot her presence altogether, and then realised, on hearing her spoken to, that she was in the room. He never thought of her, and seldom addressed her. When he did, he called her Edith, as he heard his sister do, while she always spoke of and to him as Mr. Murray, which indeed was the name by which Mrs. Lester always called him in speaking to her. What little George knew of her he liked. She was quiet and gentle, docile to her aunt, and reticent with himself. This last trait especially pleased him. Had she shown any disposition to "make friends," he would have administered one of the quiet but decided snubs which he always had at command, and have left her to digest it as she could. But no one, except a brute, could have snubbed Edith Lester, and by degrees, without his being aware of it, he grew accustomed to, and then mildly glad of, her presence in the house. She was something young, and now and then it flashed upon him that her life must be very dull spent with two quiet people like his sister and himself. Still he never took any notice of her until one day in the winter, and then it happened quite spontaneously, and almost before he knew what he was doing.

Coming unexpectedly, and without even knocking at the door, into his sister's private sitting-room, he heard her saying in tones the reverse of amiable;

"I am sure you need not complain, Edith; be thankful that you have a good home, and don't be always wishing for amusement."

He paused involuntarily, for the words jarred upon him, and as neither of the ladies saw him standing behind the screen, he heard Edith's reply:

"I am not complaining, aunt, I am only a little disappointed; I should have enjoyed seeing the flowers"

"Well, my dear," said her aunt briskly, in that cheerful tone in which some people cut off the pleasures of others, "you will have to make up your mind not to go. I can't take you, and that is all about it."

"What is it?" asked George, coming forward; his deep masculine voice striking in upon his sister's irritated treble, and Edith's low, depressed tones.

Both ladies looked up in astonishment, and Edith blushed deeply and said nothing, but Mrs. Lester at once began to justify herself.

"It is the flower show, the chrysanthemum show, this afternoon. I had said something about taking Edith, and the silly child took it for granted we were going. Now my cold is so bad I cannot go out, and this ungrateful girl grumbles at her disappointment, instead of sympathising with me and doing what she can for me."

Even Edith's mild spirit was raised by this unjust and ungenerous attack.

"Oh, aunt," she said, in a voice which was rather unsteady, "I did not grumble, and I am not ungrateful; I only said I was sorry."

George turned and looked at her with his keen eyes. He was struck suddenly with the exceeding prettiness of the delicate, flushed face, and looked at it until Edith, already alarmed at having spoken so boldly, was feeling that he must be filled with horror at her behaviour, and turned away her head to hide her painfully rising-colour and tremulous lips.

"The chrysanthemum show!" he repeated. "I have got a ticket for that. I had not intended to go, but now—Are you very anxious to see it, Edith?"

"I should have liked to go very much," she answered shyly, "if Aunt Frances could have taken me."

"Then you shall go," he said in his quiet, decided voice. "I dare say you would enjoy it; your life must be rather dull, I should think. You will trust her with me, I suppose, Frances," turning to his sister.

"Oh yes, she may go," was the rather ungracious reply; "only I am afraid she will be in your way."

"Well, I shan't tell her if she is," he rejoined, looking at the girl with a smile which seemed to change his whole face, and which Beatrice had seen more than once when he was talking to the children at the museum, although she noticed it always faded when his eye encountered hers.

Edith was almost too shy to express her gratitude; indeed, the prospect of going to the flower show with this, to her, most majestic being, had as much terror as joy for her.

Perhaps to cut short her rather incoherent thanks, George left the room, after telling her at what hour to be ready, and Mrs. Lester and her niece were again alone. Mrs. Lester hastened to improve the occasion by impressing upon Edith how deeply indebted she was to Mr. Murray for his very great kindness. Had he saved her life, or settled a handsome income on her, Mrs. Lester could not have spoken more strongly of it. She also gave her niece instructions as to her manners and deportment, which frightened her still more, and made her almost wish she was not going.

"Above all, Edith, if Mr. Murray offers you tea, or any other refreshment, be sure you decline it. The refreshments at those places are always exorbitantly dear, and Mr. Murray is already paying five shillings for your entrance, which is very handsome of him, and you must not put him to any further expense."

"No, aunt, of course not," said Edith with burning cheeks.

"Of course he may not think of it, most likely he won't but when he sees other people going into the refreshment-room, it may just occur to him to ask if you would like a cup of tea; you must remember if he does to say, 'no, thank you.'"

"Yes, aunt," said Edith with perfect docility. It did just occur to her to think that Mr. Murray might wish for a cup of tea on his own account, and to consider what line of conduct she ought to pursue under such circumstances, but these speculations made her almost dizzy with uncertainty and apprehension, and she resolutely turned her mind from them.

"Another thing," went on her aunt, "you must not express any preferences, but do exactly what Mr. Murray tells you, and try not to be any more trouble to him than you need be."

Edith said nothing. She stood in far too great awe of her aunt to venture on justifying herself twice in one afternoon, but the feelings which filled her now were mingled. She had lost all pleasure in the thought of the flower show, and would now have preferred to stay at home and spend the afternoon in reading to her aunt to venturing out under the awful patronage of Mr. Murray. At the same time she was harbouring a rebellious feeling against her aunt. No doubt she was simple and ignorant, unused to society and its ways; still, she was neither a child nor an idiot, she had neither wish nor intention so to behave herself as to attract attention. She could walk quietly by her companion's side, admire the flowers, and speak in her naturally low and sweet-toned voice—all these things her natural instinct told her to do. She needed no instructions, and she was vexed with her aunt for supposing that she did. Then her own common sense told her that Mr. Murray, having asked her to go with him, would be bound in courtesy to behave to her as any gentleman to any lady, and to consult her wishes from time to time. She kept these reflections to herself; even had she wished she dared not have spoken them out to her aunt, but perhaps they were all the stronger for being repressed, and although neither guessed it, on that afternoon Mrs. Lester has sown a seed which, in the process of time, was to bring forth its full crop of pain, and sorrow, and trouble.

George had told her to be ready at half-past two, and punctually at that hour she left her bed-room and came downstairs. She had taken great pains with her dress, having spent most of her time in her room after escaping from Mrs. Lester and her lecture. She had brushed her gown and coat, examined her gloves, and perked up the ribbon bows on her hat, yet when she was ready she could only sigh at her reflection in the glass. None of her clothes were either new or fresh, and she was painfully conscious that a not very severe critic might have truthfully called her shabby. She had done her best with her skilful fingers with the very indifferent materials at her command, and being young, delicately made, and graceful in her movements, with the further advantage of an undeniably pretty face, the result was as attractive as it could be; but she knew that even then her appearance left something to be desired.

She was first downstairs, but George came into the room a moment afterwards. He gave her a quick look from head to foot, and his face took a momentary, though, to do him justice, quite unconscious expression, which made the girl feel perfectly miserable. But it was gone almost as soon as she saw it, and he cheerfully suggested that they should start.

During the drive to the flower show very little was said, the noise in the streets made conversation almost impossible, and Mr. Murray, too, seemed, as the Quakers say, "fastened to silence." He was thinking partly of Edith's dress. He knew nothing about a woman's wardrobe, but he had a vague general idea that a young girl's toilette should invariably be perfectly fresh and dainty, and Edith's was not. He had seen that at a glance, and he was wondering, without any of the contemptuous and slighting thoughts which she imagined filled his mind, why it was so. He knew his own wealth, he knew that his sister was rich, he knew the way in which they lived, and he wondered why Edith's dress was not in keeping with these things. Was the fault hers? He stole a glance at her, but saw at once that her clothes were put on with exquisite neatness, and he at once exonerated her from blame, but resolved that all this should be changed.

In half an hour they had reached the flower show, and as Edith saw the crowds of people, and heard the invigorating strains of the military band, her spirits began to rise, and she lost all recollection of her little shabby gown in her wish to enjoy herself.

*(To be continued.)*

